# Efficient light emission from inorganic and organic semiconductor hybrid structures by energy-level tuning

**DOI:** 10.1038/ncomms7754

**Published:** 2015-04-15

**Authors:** R. Schlesinger, F. Bianchi, S. Blumstengel, C. Christodoulou, R. Ovsyannikov, B. Kobin, K. Moudgil, S. Barlow, S. Hecht, S.R. Marder, F. Henneberger, N. Koch

**Affiliations:** 1 Institut für Physik & IRIS Adlershof, Humboldt-Universität zu Berlin, Brook-Taylor-Straße 6, 12489 Berlin, Germany; 2 Helmholtz Zentrum Berlin für Materialien und Energie GmbH, Albert-Einstein-Straße 15, 12489 Berlin, Germany; 3 Institut für Chemie & IRIS Adlershof, Humboldt-Universität zu Berlin, Brook-Taylor-Straße 2, 12489 Berlin, Germany; 4 School of Chemistry and Biochemistry and Center for Organic Photonics and Electronics, Georgia Institute of Technology, 901 Atlantic Drive, Atlanta, Georgia 30332-0400, USA

## Abstract

The fundamental limits of inorganic semiconductors for light emitting applications, such as holographic displays, biomedical imaging and ultrafast data processing and communication, might be overcome by hybridization with their organic counterparts, which feature enhanced frequency response and colour range. Innovative hybrid inorganic/organic structures exploit efficient electrical injection and high excitation density of inorganic semiconductors and subsequent energy transfer to the organic semiconductor, provided that the radiative emission yield is high. An inherent obstacle to that end is the unfavourable energy level offset at hybrid inorganic/organic structures, which rather facilitates charge transfer that quenches light emission. Here, we introduce a technologically relevant method to optimize the hybrid structure's energy levels, here comprising ZnO and a tailored ladder-type oligophenylene. The ZnO work function is substantially lowered with an organometallic donor monolayer, aligning the frontier levels of the inorganic and organic semiconductors. This increases the hybrid structure's radiative emission yield sevenfold, validating the relevance of our approach.

## Abstract (web summary)

Hybrid inorganic-organic structures can overcome the limits of inorganic semiconductor light emitting devices but the energy level offset is an obstacle. Here, Schlesinger *et al.* lower the ZnO work function with an organometallic donor monolayer and enhance the radiative emission of the hybrid structure.

## Graphical abstract

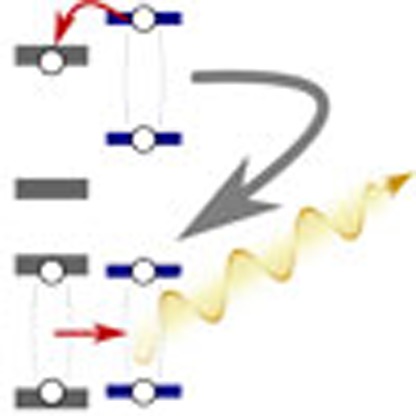

Semiconductor light emitters are an inherent part of our modern information technologies. The use of heterostructures combining different semiconductor materials has enabled vast progress, for example, for displays in terms of power consumption, colour range and brightness. Control and engineering of the hetero-interface is the key for achieving improved device performance or even new functionalities. Here, we escalate the meaning of ‘hetero' by focusing on hybrid structures, which combine inorganic and organic semiconductors (HIOS). The major advantage arising from this combination is that complementary favourable features of these material classes can be utilized for producing light. Inorganic semiconductors exhibit extended band-states and high charge carrier mobility, and large densities of electron-hole pairs can be generated by electrical injection. The electronic states of organic semiconductors are localized on the molecular scale providing strong light-matter coupling and, in some cases, a radiative yield close to unity. Moreover, the versatility of chemical synthesis allows for optimizing organic material properties, for example, tuning of the emission wavelength. All these characteristics predestine the organic semiconductor for the role of the light emitter, while its inorganic counterpart is exceptionally suited as excitation source. Importantly, electrons and holes need not be transported separately across the hybrid interface, but resonant energy transfer can directly connect the inorganic and organic exciton states, which, in a coherent regime, might even lead to the formation of hybrid excitons. Inspired by this synergistic route of function sharing, previous work has addressed and validated various theoretical and experimental aspects of HIOS, such as the presence of efficient energy transfer, however, without truly demonstrating the superior potential for light emission^1^,^2^,^3^,^4^,^5^,^6^,^7^,^8^.

From a materials perspective, ZnO is particularly well suited for this purpose because it is abundant, energy-efficient, cheap and non-toxic, and its interfacing with organic semiconductors is an emerging research topic, also in relation to photovoltaic applications^1^,^2^,^9^,^10^,^11^,^12^,^13^,^14^,^15^. This is further justified by the high structural quality of ZnO, even when grown at low temperatures^16^, thus facilitating energy efficient fabrication, as well as strong excitonic correlation (exciton binding energy of 60 meV)^17^,^18^,^19^. Efficient coupling of inorganic and organic excitons demands spectral matching of their energies. Here, we chose a triply spiro-annulated ladder-type quarterphenyl (L4P-sp3, chemical structure see Fig. 1c), which also exhibits superior photophysical properties as compared with non-rigidified oligo phenyls^20^. Its S_0_–S_1_ transition with prominent vibronic features covers the exciton resonance region of ZnO, a small Stokes shift facilitates energy migration within the organic layer, and the quantum yield of emission is high. Although the ZnO/L4P-sp3 HIOS appears ideally suited so far, a severe limitation originates from the fact that L4P-sp3 ionization energy and electron affinity, as of most organic semiconductors, is low compared with the respective values of ZnO and other traditional wide-bandgap inorganic semiconductors. Therefore, instead of the target alignment in Fig. 1a, a type-II discontinuity as shown in Fig. 1b is to be expected, where, subsequent to energy transfer, charge separation occurs at the interface, which leads to a strong quenching of the luminescence from the organic. Indeed, we observe a photovoltaic response of heterojunctions formed by ZnO with several organic semiconductors that exhibit an interfacial energy level alignment as depicted in Fig. 1b. Aligning the frontier energy levels to switch off charge separation simply by molecular design is difficult. A substantial lowering of the ZnO work function (*Φ*) by >1 eV could be one way to arrive at the situation depicted in Fig. 1a. Consequently, inserting appropriate interlayers comprising dipoles between inorganic and organic layers to shift the electrostatic potential emerges as a natural way to tune the HIOS energy level alignment. Lowering the work function of ZnO using dipolar self-assembled monolayers attached to ZnO has met with only moderate success; the lowest values of *Φ* achieved have been in the range of 3.5–4.3 eV (refs 21, 22), which is insufficient to eliminate energy level offsets with most organic semiconductor materials.

An alternative way to lower (increase) *Φ* of ZnO is the insertion of a monolayer of strong molecular donors (acceptors), which engage in charge transfer with ZnO and thus modify the surface electrostatic potential, which then shifts the energy levels of a subsequently deposited organic semiconductor accordingly. In fact, only one example of increasing *Φ* of ZnO was demonstrated so far, using perfluorinated tetracyanoquinodimethane (F4TCNQ) as an electron acceptor^23^. In this case, the *Φ* increase was assisted by upward surface band bending within ZnO. In fundamental contrast, decreasing *Φ* of ZnO with a donor is way more challenging, as shown in this work, as band bending cannot contribute notably. A molecular donor that has recently been introduced as n-dopant for organic semiconductors, including studying of trap-filling in C_60_, due to its low electron affinity is [RuCp*mes]_2_ (chemical structure see Fig. 1d)^24^,^25^,^26^,^27^.

Here we report that, employing this donor, which is sufficiently stable to be handled in air, we have achieved *Φ* values of ZnO as low as 2.2 eV, thus even rivalling those of alkali metals. This approach allows us to achieve almost perfect alignment of the frontier levels of ZnO and of L4P-sp3, thereby drastically enhancing the radiative efficiency of the HIOS.

## Results

### Interface energy levels

First, we show, with ultraviolet and X-ray photoelectron spectroscopy (UPS and XPS, respectively), that [RuCp*mes]_2_ is capable of reducing *Φ* of two different ZnO surfaces, that is, Zn-terminated ZnO(0001) and O-terminated ZnO(000-1) (for properties of the clean surfaces in UHV see ref. 23). Evaporating approximately one molecular layer of [RuCp*mes]_2_ on ZnO(000-1) reduces *Φ* by 1.5 eV to a value of 2.7 eV (see Fig. 2). For ZnO(0001), with an initial *Φ* of 3.7 eV, incremental [RuCp*mes]_2_ deposition also lowers *Φ*, ultimately reaching even 2.2 eV at approximately monolayer coverage (Fig. 2). Notably, the valence region of ZnO/[RuCp*mes]_2_ (Fig. 2) does not feature detectable photoemission intensity in the ZnO bandgap. This implies that the [RuCp*mes]_2_ interlayer does not induce a large gap-state density that could act as exciton quencher at the interface.

The origin of the *Φ* decrease is attributed to electron transfer from the interlayer to ZnO. It has been shown that [RuCp*mes]_2_ and related dimers^28^ react with organic acceptors to form two acceptor anions and two monomeric cations, in this case, [RuCp*mes]^+^ (refs 25, 26). XPS data in the C 1s and Ru 3d core level regions are consistent with the formation of [RuCp*mes]^+^ on ZnO (Fig. 2). On ZnO(000-1) two distinct Ru 3d_5/2_ peaks (the Ru 3d_3/2_ peaks are obscured by the C 1s peaks) are observed, providing evidence of two differently charged Ru species. Although the formal oxidation state of Ru is the same (2+) in both dimer and monomeric cations, the presence of the positive charge decreases the electron density in the Ru valence levels of [RuCp*mes]^+^, which screens the core potential less efficiently and gives rise to a core-level shift to higher binding energy (BE); thus, the low BE peak is attributed to excess neutral (unreacted) [RuCp*mes]_2_ and the high BE component to [RuCp*mes]^+^. This assignment is in line with that made for a similar Rh-based metal-organic complex deposited on graphene^27^. On ZnO(0001) only the 3d_5/2_ peak assigned to [RuCp*mes]^+^ is observed. The presence of [RuCp*mes]_2_ on ZnO(000-1) may arise from excess dimer (multilayer). Alternatively, due to the different saturation *Φ* achieved for the Zn- and O-terminated surfaces, a different surface electrostatic potential landscape emerges, which could impact the area-density of charged complexes and lead to the coexistence of neutral and cationic species^29^.

It is noteworthy that the *Φ* change solely originates from the localized dipole formed by the [RuCp*mes]^+^ and the negatively charged ZnO as a result of the charge-transfer reaction. Contributions due to surface band bending are absent, as evidenced by the constant binding energy of Zn 3p and O 1s core levels even after deposition of [RuCp*mes]_2_ (see Supplementary Fig. 1). There is, however, a small shift of the surface-hydroxy O 1s component to lower BE by ∼0.2 eV; we attribute this to an enhanced core hole screening arising from the immediate proximity of the interlayer and the increased electron density at the ZnO surface. This is fundamentally different from the situation of molecular acceptor interlayers, where a significant (up to 0.8 eV) upward band bending in ZnO was observed^23^. However, this can easily be rationalized by the n-doped nature of ZnO. While molecular acceptors deplete the ZnO-native donor levels, molecular donors fill the ZnO conduction band. The orders-of-magnitude larger density of states of the conduction band, as compared with that of the native donors, strongly pins the Fermi level (*E*_F_) as soon as the ZnO turns degenerate.

Next, we assess the energy level alignment of bare and interlayer-covered ZnO with the organic semiconductor L4P-sp3. The valence band maxima of bare ZnO(0001) and ZnO(000-1) are at 3.4 and 3.0 eV below *E*_F_ (Fig. 3), respectively, implying slight downward surface band bending for ZnO(0001) and upward for ZnO(000-1). Deposition of L4P-sp3 reduces *Φ* of both surfaces by 0.2 eV; this is often observed for organic materials physisorbed on metal oxides and attributed to a mechanism analogous to ‘push-back' on metal surfaces^30^,^31^. No new core level peaks arise in the O 1s and Zn 3p regions, thus evidencing physisorption of L4P-sp3. Furthermore, there are no L4P-sp3 thickness-dependent spectral changes (up to 50 Å nominal coverage, that is, multilayer). Hence, the HIOS energy level alignment is of type-II with an offset between the respective filled/empty frontier levels of 1.1 eV [ZnO(0001)] and 1.2 eV [ZnO(000-1)] (see Fig. 4), that is, favourable for charge separation yet unfavourable for light emission.

On [RuCp*mes]^+^-covered ZnO(000-1), which, as noted above, has a work function of 2.7 eV, L4P-sp3 deposition does not induce a *Φ* change. The onset of emission from the level associated with the highest occupied molecular orbital (HOMO) of L4P-sp3 is 3.1 eV below *E*_F_ (Fig. 3b). On the [RuCp*mes]^+^-covered ZnO(0001) surface, with the lower *Φ* of 2.2 eV, L4P-sp3 deposition increases *Φ* to 2.5 eV (Fig. 3a). From this observation and the fact that the HOMO onset is 3.3 eV below E_F_, in conjunction with the optical gap of 3.25 eV, one concludes that the level associated with the lowest unoccupied molecular orbital (LUMO) of L4P-sp3 is pinned at *E*_F_ (Fig. 4). However, this estimate ignores the effects of exciton-binding energy. Although the transport gap of L4P-sp3 is yet unknown^32^, it is likely to be somewhat larger than the optical gap so that the actual LUMO level distribution is at and above *E*_F_ (ref. 33). As *Φ* remains very low for both surfaces, no changes in the ZnO band bending occur. The UPS spectral signature of L4P-sp3 with and without [RuCp*mes]^+^ interlayer is the same (Fig. 4), so that the organic semiconductor levels are rigidly shifted in energy with respect to those of the inorganic component due to the interlayer. Consequently, the interlayer has aligned the energy levels of our HIOS (Fig. 4), with an offset as little as 0.1 eV. For efficient energy transfer, however, the conduction band minimum should also be in resonance with the LUMO level. We assume that the L4P-sp3 transport gap (optical gap plus exciton binding energy) is only slightly wider (< 0.3 eV) than its optical gap because of the rigidified phenyls, which lowers the exciton binding energy compared with torsionally more flexible *para*-phenyls, so that the offset between the unoccupied frontier levels of the HIOS is estimated to be similar to that measured for the occupied levels.

Notably, we observed that exposure of a sample consisting of a 5nm thick L4P-sp3 film on [RuCp*mes]^+^/ZnO(000-1) to a nitrogen glove box atmosphere for 10 min changed the interface energy levels by <0.1 eV. Therefore, these optimized HIOS structures are remarkably robust with respect to handling outside of ultrahigh vacuum conditions, which renders them of high practical relevance. Going beyond ZnO, with [RuCp*mes]^+^ we achieved a similarly low *Φ* of 2.3 eV (and 2.7 eV after air exposure) for indium-tin-oxide (ITO), which is widely used as transparent electrical contact. With our method, ITO can now form ohmic contacts for electron injection with essentially any organic semiconductor, a massive virtue in its own.

### Energy transfer and radiative recombination

**Figure 1:**
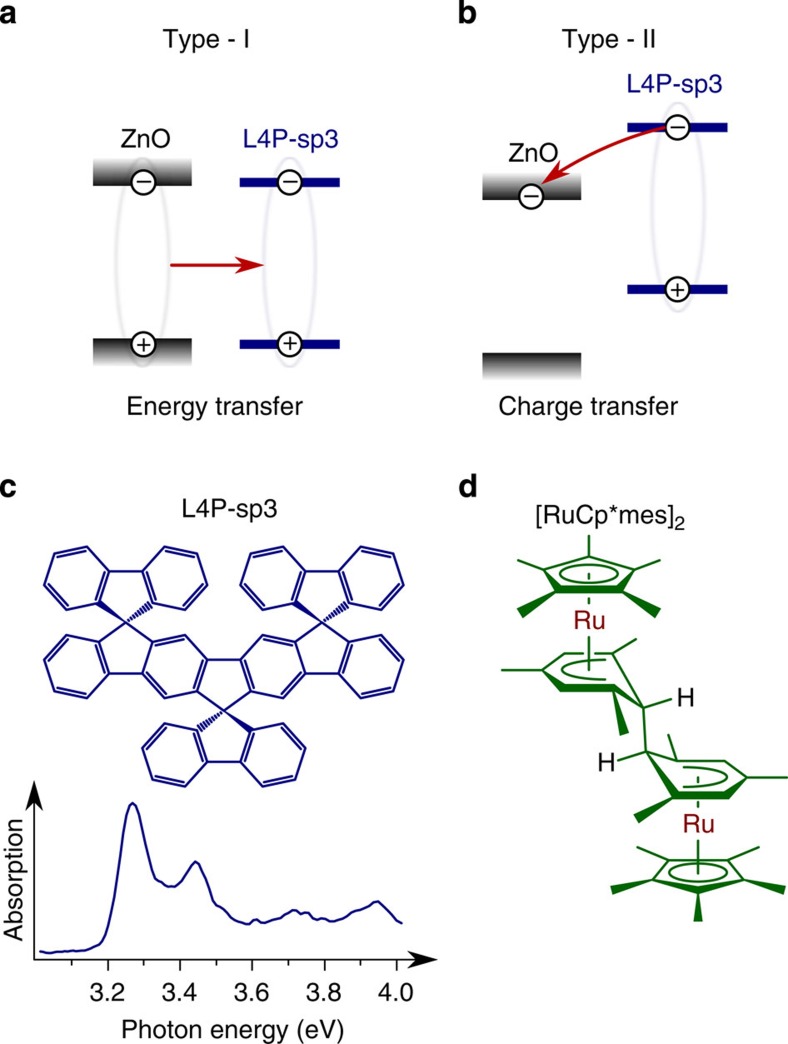
Types of energy level alignment and chemical structures. Energy level alignment favourable for (**a**) energy transfer and subsequent light emission and (**b**) exciton dissociation and charge separation. Chemical structures of (**c**) L4P-sp3 and (**d**) the donor ruthenium pentamethylcyclopentadienyl mesitylene [RuCp*mes]_2_. The spectrum in (**c**) shows the absorption spectrum of a L4P-sp3 thin film.

**Figure 2:**
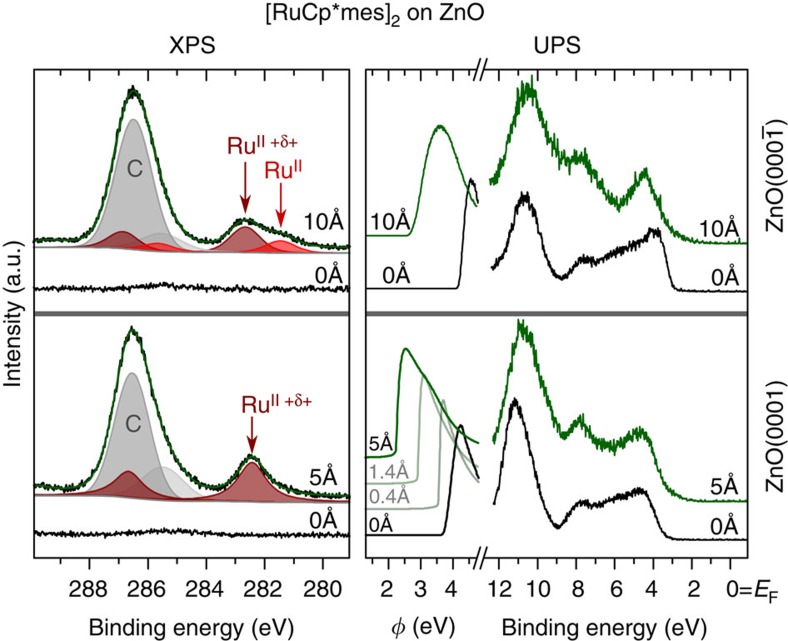
Core and valence electron spectra for [RuCp*mes]^+^ on ZnO. Photoemission spectra for [RuCp*mes]_2_ deposited on O- (*top panels*) and Zn-terminated (bottom panels) ZnO. The left side panels show the C 1s and Ru 3d core level regions. The two different Ru 3d_5/2_ peaks (top left panel) correspond to neutral [RuCp*mes]_2_ dimer with Ru in (+2) oxidation state (Ru^II^) and to the cationic [RuCp*mes]^+^ with formal Ru oxidation state (+2) but lower electron density in the valence levels (Ru^II+δ+^). The secondary electron cutoff (providing the work function *Φ*) and valence spectra are shown in the right side panels. Black curves: bare ZnO. Green curves: after deposition of [RuCp*mes]^+^ (nominal mass-thickness given in Å).

**Figure 3:**
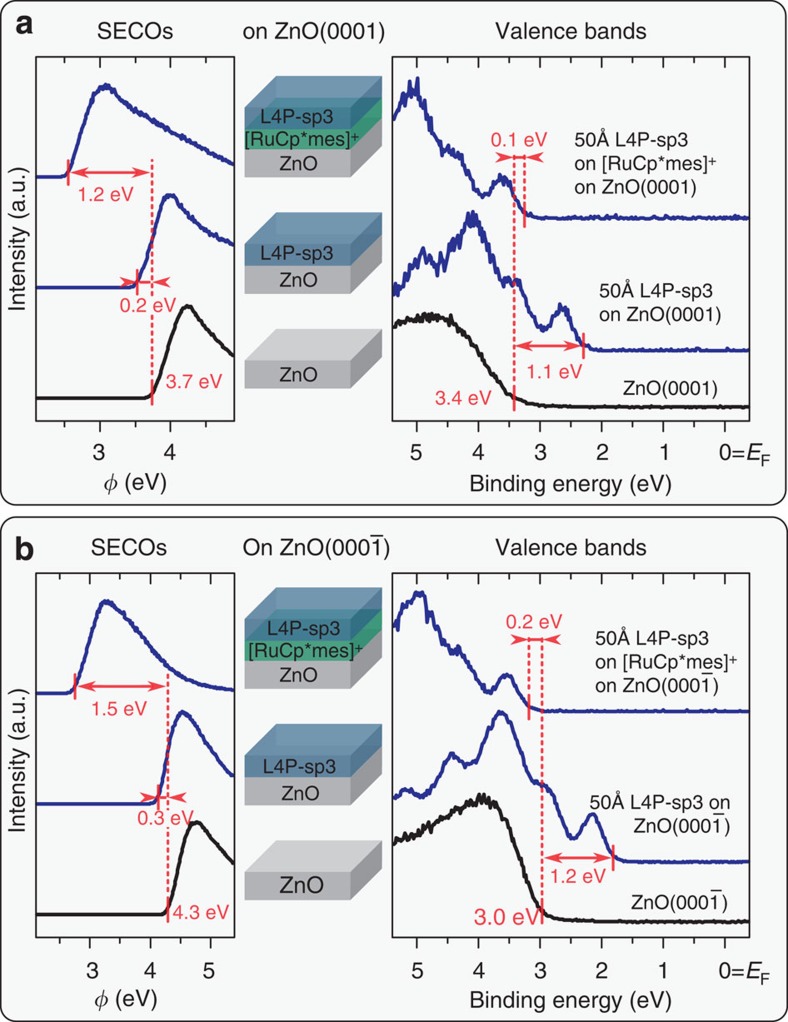
HIOS energy levels with and without [RuCp*mes]^+^ interlayer. UPS spectra of L4P-sp3 (blue curves) and ZnO (black curves). The effect of the interlayer is demonstrated on (**a**) Zn-terminated and (**b**) O-terminated ZnO. The left panels show the work function, the right panels show the valence spectra. The corresponding HIOS structure (with/without interlayer) is indicated between the panels.

**Figure 4:**
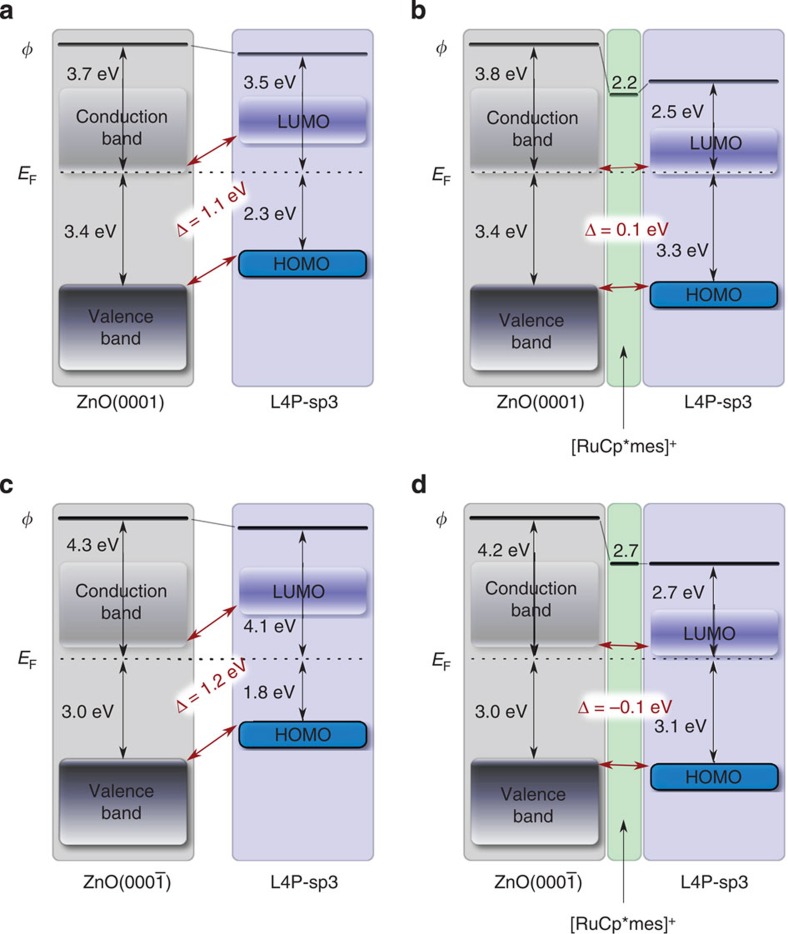
Energy level diagrams. L4P-sp3 without interlayer on (**a**) Zn-terminated ZnO(0001) and (**c**) O-terminated ZnO(000-1), and with [RuCp*mes]^+^ interlayer (**b**,**d**), respectively. Energy values are referenced to the Fermi level and in eV. The offset between the L4P-sp3 and ZnO energy levels is highlighted in red. The L4P-sp3 LUMO region is shaded with a gradient to represent uncertainties due to the unknown transport gap.

Next, we demonstrate the consequences of the energy level optimization for the balance of energy transfer versus interfacial charge separation at the hybrid interface (Fig. 1). The inorganic part for this study consists of a ZnO/Zn_0.9_Mg_0.1_O quantum well (QW) structure grown by molecular-beam epitaxy^34^. The role of the QW is to collect the electron-hole pairs injected into the inorganic component. The confinement-induced shift of the exciton energy in the 3.5-nm-wide ZnO QW is only in the 10 meV range and thus of no relevance in the present context. A thin (2 nm) Zn_0.9_Mg_0.1_O top barrier assures that electron-hole pairs in the QW are close enough to the L4P-sp3 organic layer for subsequent energy transfer, but avoids direct electronic coupling with the molecular states. The selected Mg content of 10% shifts the CBM (VBM) by 0.16 eV (0.06 eV) to higher (lower) energies as compared with ZnO (refs 35, 36). This gap-widening of the top barrier is very small compared with the much larger inorganic/organic type-II level offset, and charge separation leading to luminescence quenching is also expected for the pristine Zn_0.9_Mg_0.1_O/L4P-sp3 interface. When [RuCp*mes]^+^ is inserted, the QW and the L4P-sp3 energy levels are essentially the same as in Fig. 4d, that is, well aligned, thus we also expect efficient energy transfer from the QW structure and subsequent light emission from L4P-sp3.

**Figure 5:**
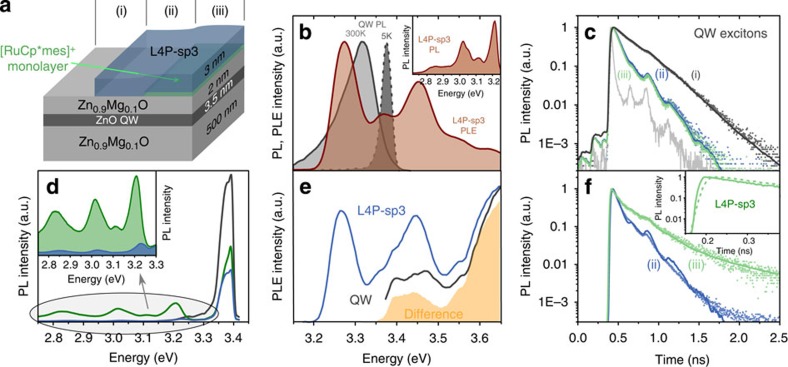
Optical spectra of HIOS. (**a**) Schematics and colour code of the investigated structures: grey (i) bare ZnO/Zn_0.9_Mg_0.1_O QW structure, blue (ii) HIOS consisting of the QW structure overgrown with 3 nm L4P-sp3, green (iii) HIOS with [RuCp*mes]^+^ interlayer between QW structure and L4P-sp3. (**b**) Emission and absorption of the isolated individual components. (**c**) PL decay transients of the QW excitons (*T*=5 K) of structures (i), (ii) and (iii). Light grey: instrumental response of the time-correlated single-photon counting set-up on the fs excitation pulse. Only the QW is primarily excited. Solid curves are fits to the data by convoluting exponential transients *N*(*t*) (with up to three components) with the instrumental response function *IRF(t')* according to 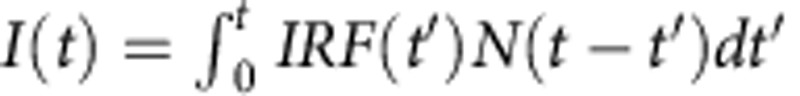. Lifetimes given in the text are time averages over these decay curves 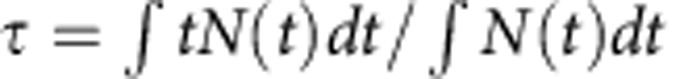. (**d**) PL spectra of structures (i), (ii) and (iii). (**e**) PLE of optimized structure (iii) (detection at the S_1,ν=0_–S_0,ν=1_ emission peak of L4P-sp3 and low energy side of the QW emission). Yellow: difference spectrum of L4P-sp3 in (iii) and on sapphire. (**f**) L4P-sp3 PL transients of (ii) and (iii). The excitation photon energy (3.3 eV) is below the ZnO absorption edge, excluding contributions from energy transfer. Evaluation as in (**c**). Inset: transients of (iii) right after excitation demonstrating the presence of a rise time (dashed curve) for an excitation energy (3.46 eV) above the ZnO optical gap.

Using shadow masks during molecular deposition, three types of structures were prepared on the same wafer (Fig. 5a): bare ZnO/Zn_0.9_Mg_0.1_O QW structure [structure (i)], 3 nm L4P-sp3 film on the QW structure [pristine HIOS, structure (ii)] and approximately monolayer [RuCp*mes]^+^ embedded between the QW structure and 3 nm L4P-sp3 [optimized HIOS, structure (iii)].

For the characterization of L4P-sp3 alone, a further reference sample comprised an upright standing monolayer of tetracontane (C_40_H_82_) between L4P-sp3 and the QW structure, where neither energy transfer nor charge separation is of relevance. Owing to its rigid backbone, L4P-sp3 exhibits a distinct vibronic progression in the S_0_–S_1_ absorption band with the dominant S_0,ν=0_-S_1,ν=0_ transition practically in resonance with the room temperature photoluminescence (PL) band of the QW (Fig. 5b). Therefore, the extensive spectral overlap between emission of the donor (QW) and absorption of the acceptor (L4P-sp3) needed for efficient energy transfer is assured. The emission of L4P-sp3 mirrors the absorption progression with a Stokes shift of 40 meV. The L4P-sp3 lifetime derived from the PL decay transients with the tetracontane spacer is *τ*_*m*_=500 ps and agrees well with data for thick films on sapphire.

The interplay of energy and electron transfer across the HIOS is elucidated by PL excitation (PLE) and time-resolved PL (TRPL) measurements at low temperature (*T*=5 K). Here the PL of the QW is blue-shifted resulting in less spectral overlap with the L4P-sp3 absorption compared with that at room temperature (Fig. 5b), but a markedly smaller spectral width provides for better separation of the individual processes by selective excitation. The lifetime of the QW exciton shortens from *τ*_QW_=205 ps in the bare structure to 
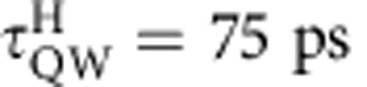
 in the pristine HIOS, demonstrating opening of an extra decay channel (Fig. 5c). Using 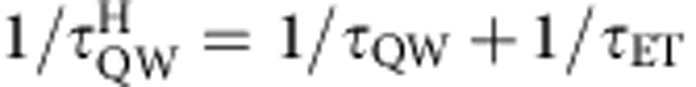 we find an energy transfer time of *τ*_ET_=115 ps or an efficiency of 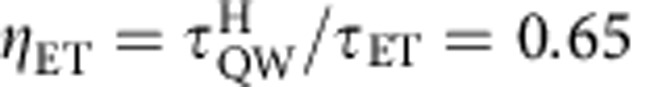. These values are consistent with the drop of the QW emission from structure (i) to structure (ii) (Fig. 5d). However, this drop does not translate into a corresponding increase of the L4P-sp3 emission (Fig. 5d). In line with this finding, we observe a one-order-of-magnitude shorter molecular lifetime 
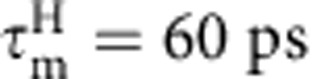
 in structure (ii) (Fig. 5f).

The low emission yield and the short lifetime are a direct result of the type-II energy level alignment of pristine HIOS (see Fig. 4a,c). Efficient energy transfer alone requires solely spectral overlap between the PL spectrum of the QW donor and the absorption spectrum of the molecular acceptor. As this condition is fulfilled in HIOS (ii), QW excitons are efficiently converted into Frenkel excitons of L4P-sp3. However, once the excitons are transferred to the organic layer, they are rapidly quenched due to charge separation at the Zn_0.9_Mg_0.1_O/L4P-sp3 interface and thus the overall luminescence yield of HIOS (ii) is very low. This type of hybrid structure is thus unsuited for light-emitting applications. In the thin (3 nm) L4P-sp3 layer used here, diffusion of excitons towards the interface is not an important factor. Thus, the characteristic time and efficiency of the charge separation process can be estimated by 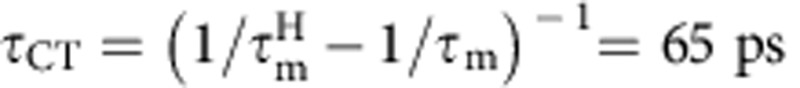 and 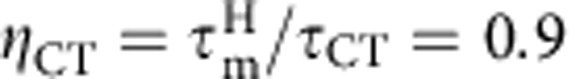, respectively. That is, 9 out of 10 excitons generated (directly or indirectly) in L4P-sp3 are not converted into emitted light. The [RuCp*mes]^+^ interlayer in structure (iii) alters the scenario profoundly. The L4P-sp3 lifetime recovers to a value of 
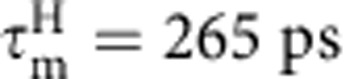
 (Fig. 5f) signifying considerable suppression of exciton quenching. Fully consistent with the change in lifetime, the molecular emission increases by a factor of seven compared with structure (ii) (Fig. 5d). Substantial energy transfer from the QW to L4P-sp3 in structure (iii) is clearly demonstrated by PLE. The spectrum taken at the S_1,ν=0_–S_0,ν=1_ line of the molecular emission clearly shows the absorption features of the QW as well as that of the Zn_0.9_Mg_0.1_O band gap edge (> 3.55 eV), more obviously so when the difference spectrum with respect to L4P-sp3 on sapphire is constructed (Fig. 5e). Further evidence is provided by the presence of a rise time in the L4P-sp3 emission (now resolvable due to the longer decay time) displaying the arrival of the excitons in the organic layer (inset Fig. 5f). The lifetime of the QW excitons in structure (iii) is not markedly changed, though the donor-acceptor spatial separation is widened by ∼0.3 nm thickness of the [RuCp*mes]^+^ interlayer. In addition to opening of the energy transfer channel, the presence of [RuCp*mes]^+^ and L4P-sp3 is also likely to change the electrostatic environment of the QW and, hence, the electron-hole separation of the exciton, which determines its lifetime. Hence, 
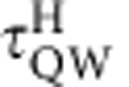
 in structure (ii) and structure (iii) cannot be compared directly. However, for the optimized structure (iii), all data consistently yield an efficiency of *η*_ET_=0.65. The yield of photons emitted by the L4P-sp3 layer per electron-hole pair generated in the QW (either by optical or electrical excitation) is *η*=*η*_ET_*η*_PL,L4P–sp3_. The latter quantity is the emission yield of L4P-sp3 in the hybrid structure. As there is still residual exciton quenching at the ZnMgO interface, *η*_PL,L4P–sp3_≈0.55, assuming that the intrinsic PL yield of L4P-sp3 approaches unity. Hence, the total luminescence yield of HIOS (iii) is η≈0.35.

At room temperature, the role of the [RuCp*mes]^+^ interlayer is even more crucial. Whereas L4P-sp3 emission from structure (ii) is no longer detectable, the signal remains bright in the case of structure (iii). Despite this impressive improvement of radiative emission yield, particularly at room temperature, the present HIOS can further be optimized, since a considerable fraction of excitons is still not used for light emission. This might be traced back to interface states, which are too low in intensity to be directly revealed by photoemission. To further optimize such HIOS, current work focuses on identifying and avoiding non-radiative side channels as well as on routes to increase *η*_ET_. The latter could be improved by employing non-polar QW structures, as here piezoelectric fields are avoided, leading to an enhanced electron-hole wave function overlap and a concomitant increase of the transition matrix element mediating energy transfer.

## Discussion

We have introduced a simple method to tune the energy level alignment at hybrid inorganic/organic semiconductor structures. The work function of the inorganic HIOS component (exemplified for ZnO and ITO) is lowered to a record value of 2.2 eV by depositing the organometallic donor dimer [RuCp*mes]_2_, which adopts the cation monomeric [RuCp*mes]^+^ form in contact with the oxide surfaces. With [RuCp*mes]^+^, the subsequently deposited organic component (L4P-sp3) then realigns its energy levels with respect to those of ZnO such that efficient energy transfer and radiative recombination is enabled. It is perceptible that the use of such molecular interlayers is generally applicable to optimize the energy levels in a wide range of HIOS, especially with wide-gap inorganic semiconductors. Although further optimization is needed, our demonstration of a sevenfold increase of the radiative emission yield in a HIOS, in combination with high ambience stability, substantiates the huge potential of such structures in light-emitting applications that demand high frequency response and broad spectral ranges, particularly for upcoming information display and imaging technologies and ultrafast data processing and communication.

## Methods

### Photoemission measurements

Photoemission experiments were performed at the SurICat end station of beamline PM4 at BESSY II and at a custom-built UHV chamber, both allowing transfer between preparation (base pressure 2 × 10^−8^ mbar and 5 × 10^−10^ mbar) and analysis chamber (base pressure 2 × 10^−10^ mbar and 1 × 10^−10^ mbar) without breaking UHV conditions. Excitation energies were 35 and 620 eV (synchrotron), and the He I line of a gas discharge lamp as well as the Al K_α_ line in the lab. Photoelectrons were detected using a Scienta SES 100 and a Specs Phoibos100 hemispherical electron spectrometer, respectively. [RuCp*mes]_2_ and L4P-sp3 were evaporated from resistively heated quartz crucibles onto bulk ZnO crystals purchased from Tokyo Denpa. Evaporation rates were in the range between 0.1 and 1 nm min^−1^. Film mass thicknesses were monitored via quartz crystal microbalances. Before performing the experiments the ZnO crystals were cleaned by repeated Ar-sputter/annealing cycles (1 kV, 2 μA, 40 min; 400 °C) and cleanliness verified by the C1s emission to be below 1%. Selected samples were exposed to air or N5 nitrogen to simulate the effects of typical glove box environments and its effect on the HIOS energy level alignment.

### Fabrication of ZnO quantum well and organic hybrids

For the optical measurements of energy transfer analogous structures were grown at a third apparatus. There ZnO quantum well structures were prepared in a molecular beam epitaxy apparatus (DCA, Finland) and organic films deposited in a home-built system afterwards, both in ultra-high vacuum (10^−9^ mbar). Film thicknesses and deposition rates (0.1 nm min^−1^) were controlled by a quartz crystal microbalance. Transfer of the samples between the chambers for organic and inorganic growth was performed at 10^−5^ mbar. Samples were then transferred to a cryostat through a glove box. The energy level alignment of those samples was checked by a control sample, which was also transferred via a glove box to the previously mentioned photoemission set-ups.

### Optical Characterization

PLE measurements were performed with a Xe lamp as excitation source combined with a double monochromator in additive configuration. The PL signal was spectrally dispersed in a 0.3-m monochromator and detected by a photomultiplier tube. PL transients were obtained by time-correlated single-photon counting using a microchannel plate photomultiplier mounted to a monochromator with an overall time resolution of 20 ps. A mode-locked frequency-doubled fs-Ti:sapphire laser (5 W cm^−2^, 76 MHz) served as an excitation source. The excitation density is chosen sufficiently low to avoid bimolecular processes. Decreasing the excitation density by a factor of 100 does not result in changes of the characteristic transfer times as well as the QW life time as shown in the supporting information (Supplementary Fig. 2).

## Author contributions

R.S., S.Bl., F.H. and N.K. coordinated the work and wrote the initial manuscript. R.S., F.B., C.C. and R.O. performed experiments. R.S. and S.Bl. analysed the data. B.K, K.M., S.Ba., S.H. and S.M. provided the organic materials. All authors discussed the results and provided input to the manuscript.

## Additional information

**How to cite this article:** Schlesinger, R. *et al.* Efficient light emission from inorganic and organic semiconductor hybrid structures by energy-level tuning. *Nat. Commun.* 6:6754 doi: 10.1038/ncomms7754 (2015).

## Supplementary Material

Supplementary Information Supplementary Figures 1-2
