# Supplementary material for: Efficient light emission from inorganic and organic semiconductor hybrid structures by energy-level tuning
Source: Nat Commun. 2015 Apr 15;6:6754. doi: 10.1038/ncomms7754 (PMC4410639; doi:10.1038/ncomms7754)
Supplement: Supplementary Information — Supplementary Figures 1-2 [file ncomms7754-s1.pdf]

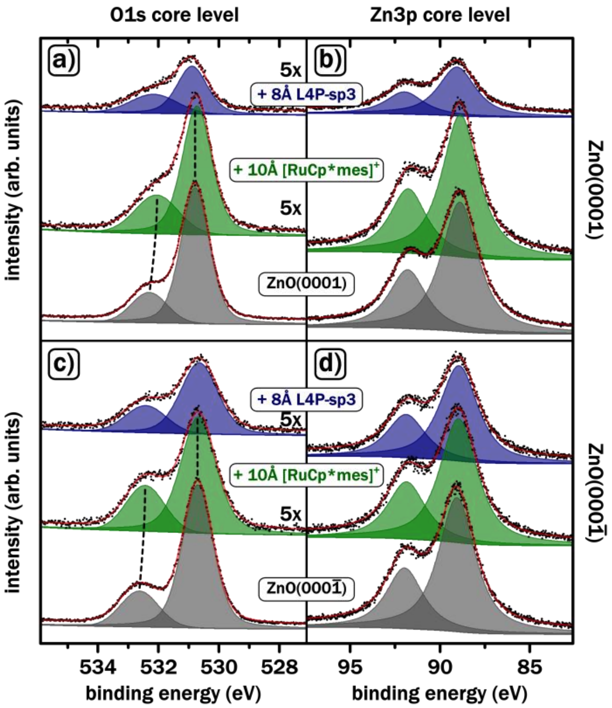

<u>Supplementary Figure 1</u> Absence of absorption induced band bending Core level photoemission of ZnO evidencing the internal band bending upon absorption of $[RuCp^*mes]^+$ (green) and L4P-sp3 (blue). a) and c) showing the O1s core level; b) and d) showing the Zn3p core level. The upper panels a) and b) show the results for ZnO(0001), while c) and d) for ZnO(000-1). Upon molecular absorption the ZnO features stay constant in position. Only the OH related peak at ~532.5 eV BE shifts closer to the bulk O peak. This is due to enhanced photohole screening from the neighboring charge transfer layer with $[RuCp^*mes]^+$.

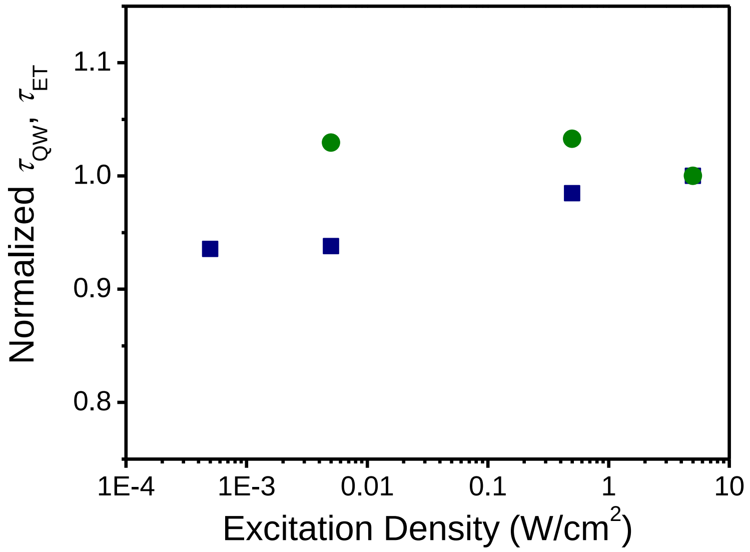

<u>Supplementary Figure 2</u> Excitation density dependence of the QW PL life time τ_{QW} in HIOS (i) (blue squares) and the energy transfer time τ_{ET} in HIOS (ii) (green circles). The characteristic times are normalized to the value measured at 5 W cm^{-2} for better visibility.
